# Assessment of Bereaved Caregiver Experiences of Advance Care Planning for Children With Medical Complexity

**DOI:** 10.1001/jamanetworkopen.2020.10337

**Published:** 2020-07-28

**Authors:** Sarah Lord, Clara Moore, Madison Beatty, Eyal Cohen, Adam Rapoport, Jonathan Hellmann, Kathy Netten, Reshma Amin, Julia Orkin

**Affiliations:** 1 Division of Paediatric Medicine, The Hospital for Sick Children, Toronto, Ontario, Canada; 2 Department of Paediatrics, University of Toronto, Toronto, Ontario, Canada; 3 Child Health Evaluative Sciences, SickKids Research Institute, Toronto, Ontario, Canada; 4 Institute of Health Policy, Management, and Evaluation, University of Toronto, Toronto, Ontario, Canada; 5 Emily’s House Children’s Hospice, Toronto, Ontario, Canada

## Abstract

**Importance:**

Advance care planning (ACP) is the process of discussing values and preferences for care to help inform medical decision-making. Children with medical complexity (CMC) often have a shortened life span with an unpredictable clinical course and timing of death; however, there is a paucity of literature that describes the experience of ACP from the perspective of bereaved family caregivers of CMC.

**Objective:**

To explore the experiences of bereaved family caregivers with ACP for CMC.

**Design, Setting, and Participants:**

This qualitative study included 12 interviews with 13 bereaved family caregivers of CMC whose deaths had occurred in the 5 years before study commencement (2013-2018). Participants were recruited at a single tertiary care pediatric center; CMC were treated by the Complex Care or Long-term Ventilation clinic in Toronto, Ontario, Canada. Data were collected from July to October 2018. Thematic analysis with an inductive approach was used.

**Exposures:**

Qualitative interviews were conducted using purposive sampling of bereaved family caregivers using semistructured interviews that were recorded and transcribed. Interviews were conducted until saturation was reached.

**Main Outcomes and Measures:**

Transcripts were analyzed to create themes that characterized caregiver experiences with ACP.

**Results:**

A total of 13 family caregivers were interviewed in 12 interviews, all of whom were parents (12 [92%] women, 1 [8%] man) of a deceased child (aged 7 months to 12 years). Themes were divided in the 3 following categories, which align with the Donabedian model for health service quality: (1) structure of care, (2) ACP process, and (3) end-of-life experience. Notable subthemes for this population included the importance of accounting for parental expertise in the child’s care, recurrent experiences with life-threatening events, relative shock of the timing of death, and the multiple losses that caregivers experienced.

**Conclusions and Relevance:**

In this study, parental experiences revealed that there are key aspects of the structure of the child’s care, process around ACP, and end-of-life care experiences that provide important reflections on ACP that warrant future study.

## Key Points

**Question:**

How do bereaved family caregivers describe their experiences with advance care planning for children with medical complexity?

**Findings:**

In this qualitative study that included 13 parents of children with medical complexity, participants emphasized the importance of involving trusted health care professionals and incorporating parental expertise to guide advance care planning. The relative shock parents experienced regarding the timing of the child’s death, despite recurrent experiences with life-threatening events, and the multiple losses they experienced when the child with a large health care team died were important themes.

**Meaning:**

The perspectives of bereaved parents in this study revealed important themes that should be considered for future study of advance care planning for children with medical complexity.

## Teaser

This qualitative study explores the experiences of bereaved family caregivers with advance care planning for children with medical complexity.

## Introduction

Medical and technological advances have resulted in a growing cohort of children with medical complexity (CMC), many of whom would not have survived previously.^1,2^ These children are often defined as having high family-identified needs, complex chronic disease necessitating specialized care, functional disability, and high health care utilization.^3^ They are among the most medically fragile pediatric patients and are at high risk of morbidity and mortality during childhood.^3,4^ Given their baseline medical and technological needs, decision-making regarding medical interventions that optimally balances quantity and quality of life is challenging.

Advance care planning (ACP) is the process of discussing values and preferences to help inform medical decision-making.^5^ In pediatrics, this often involves shared decision-making among parents and health care professionals to align care with the child’s best interests. ACP discussions are often prompted by the anticipation of impending death, including do-not-resuscitate orders, or when curative therapies are no longer effective and palliative care becomes the focus.^6^ This approach does not lend itself to optimal care when the course of illness and timing of death are highly unpredictable, as is often the case for CMC. Furthermore, health care professionals’ traditional practice of ACP at the end of life may miss important opportunities to explore families’ goals throughout the disease trajectory for CMC.

Prior studies have demonstrated that parents value ACP because of the potential for optimizing a child’s quality of life and achieving better end-of-life outcomes.^5^ Given the chronicity, unpredictable disease course, and tendency toward technology dependence for many CMC, we felt it would be worthwhile to investigate ACP experiences in this population specifically. Most studies involving bereaved parents have focused on children with disease categories that tend to have a more predictable trajectory, such as cancer, congenital heart disease, and defined genetic syndromes.^5,7,8^ While some previous studies have focused on ACP for CMC, they have examined the experiences of parents and health care professionals of living CMC.^9,10,11^ The current study was undertaken to examine the experiences of ACP for CMC from the perspective of bereaved parents. Our hypothesis was that the insights from parents who had experienced the full life and death of their CMC would provide valuable additions to the existing literature that helps guide ACP practices for this population.

## Methods

### Study Design

We conducted qualitative interviews with bereaved family caregivers of CMC at The Hospital for Sick Children, Toronto, Canada. Institutional research ethics approval was obtained from the Hospital for Sick Children. Written informed consent was obtained by a research coordinator prior to initiation of the interview. Data were collected between July and October 2018 for patients who died between 2013 and 2018. The Consolidated Criteria for Reporting Qualitative Research (COREQ) checklist for reporting qualitative studies was used to guide reporting.^23^ Parents were eligible for study participation if their child met at least 1 criterion from each of the following conditions, per the Ontario Provincial Council of Maternal and Child Health criteria for CMC^12^: (1) technology dependent and/or user of high intensity care, (2) fragility, (3) chronicity, and (4) complexity. Furthermore, their children had to have been treated in the Complex Care or Long-term Ventilation (LTV) clinic, parents had to have been the primary caregiver for a CMC and experienced the death of that child between 6 months and 5 years before the recruitment date, and parents had to have had at least 1 ACP discussion (identified by a member of their former Complex Care or LTV team). While definitions of CMC vary, these criteria have been successfully implemented for prospective recruitment to complex care programs across Ontario, Canada’s largest province (population, 14.5 million). It is estimated that as many as 0.67% of children in Ontario meet these criteria.^13^ Families were excluded if their child’s death occurred within 6 months of the recruitment date to respect their early months of bereavement and avoid any added psychological burden associated with discussing those events in the context of a research study. Parents were recruited through purposive sampling to ensure representation of CMC of various ages, diagnoses, technology supports, and experiences (positive and negative) with ACP. Criteria for ACP discussions were not strictly defined, given that the topics varied depending on the child and context. However, they shared the following characteristics: they pertained to health-related situations that might happen in the future, and they involved identifying goals of care and thinking about how to align future care with those goals.

### Data Collection

Eligible families were identified by Complex Care and LTV program physicians using their respective clinical databases. Introductory letters were sent, and follow-up phone calls were made to families who did not opt out. Data collection comprised semistructured interviews. Basic demographic information was obtained from the hospital electronic medical record.

An interview guide was developed by the research team following a review of the relevant literature^5,6,7,8,9,10,11,14,15,16,17,18,19^ and with input from pediatric complex care, LTV, and palliative care experts. All interviews were conducted in English, although interpreter services were available and offered to any family for whom English was not their preferred language. Following 2 initial interviews, transcripts were reviewed, and revisions were made to the interview guide to improve clarity (eTable in the Supplement). Interviews lasted from 40 to 80 minutes and were conducted at a location of the participant’s choice (eg, home, hospital, by telephone) by a research coordinator (C.M.) who did not have a prior relationship with the participants. Participants were compensated for their time with a nominal gift card.

### Statistical Analysis

Semistructured interviews were audio-recorded and transcribed verbatim by a professional transcriptionist. The qualitative analysis was aided by qualitative data management software (NVivo version 12 [QSR]). Four members of the research team (S.L., physician; C.M., research coordinator; M.B., research coordinator, and J.O., physician) followed the Braun and Clarke steps of thematic analysis,^20^ first by reading and rereading the data, then by the generating initial codes. Codes were organized into themes and subthemes, which were subsequently reviewed to ensure the data supported each theme. Consensus regarding codes and themes was reached through meetings among team members (S.L., C.M., M.B., and J.O.) during which emerging codes, themes, and relationships were discussed. Interviews were completed once thematic saturation was achieved.^20,21,22^ Because no statistical tests were performed, not prespecified level of significance was set.

## Results

A total of 53 eligible families were identified by the Complex Care and LTV physicians and were sent an introductory letter about the study; none of them opted out of a telephone call. Overall, 29 (55%) were unable to be reached by telephone, and 12 (23%) declined participation; thus, 12 families (23%) agreed to participate. A total of 13 bereaved family caregivers (12 [92%] mothers and 1 [8%] father) whose CMC died between 2013 and 2018 participated in the semistructured interviews. One interview was completed by both the father and the mother, while the rest of the interviews were completed by the mother alone. Demographic information regarding family caregivers and their CMC is summarized in Table 1.

**Table 1.** Demographic Information for 13 Participants and 12 Children With Medical Complexity

Characteristic	No. (%)
Parent interviewees	
Mother	12 (92)
Father	1 (8)
Child’s diagnosis type^a^	
Genetic or congenital	11 (92)
Acquired	1 (8)
Home technology supports^b^	
Feeding tube	10 (83)
Respiratory support^c^	10 (83)
Wheelchair	9 (75)
Long-term intravenous access	3 (25)
Child’s age at death, y	
<1	1 (8)
1 to <5	4 (33)
5-10	4 (33)
>10	3 (25)
Time since child’s death, y	
<1	5 (42)
1-5	6 (50)
>5	1 (8)
Palliative care team involvement	
Yes	10 (84)
No	1 (8)
Unknown	1 (8)
Location of child’s death	
Hospital	7 (58)
Hospice	5 (42)
Home	0

^a^ Because of the rare nature of these conditions, specific diagnoses were not included to maintain patient confidentiality.

^b^ Information about technology for 1 patient was incomplete.

^c^ Respiratory support included oxygen, suctioning, cough assist, tracheostomy, and ventilator support.

Following analysis and coding of the interviews, the themes were analyzed, then grouped in a way that aligns with Donabedian’s established framework for characterizing health care service quality.^24^ This model has been used as a foundation for health services research, including conceptual frameworks for communication regarding end-of-life issues, and evaluates aspects of health care according to structure, process, and outcome.^25^ The overarching themes in our study were organized as follows: (1) structure of care, (2) ACP process, and (3) end-of-life care outcomes, with subthemes grouped accordingly (Figure). Examples of quotations for each theme and subtheme are shown in Table 2.

**Figure.**
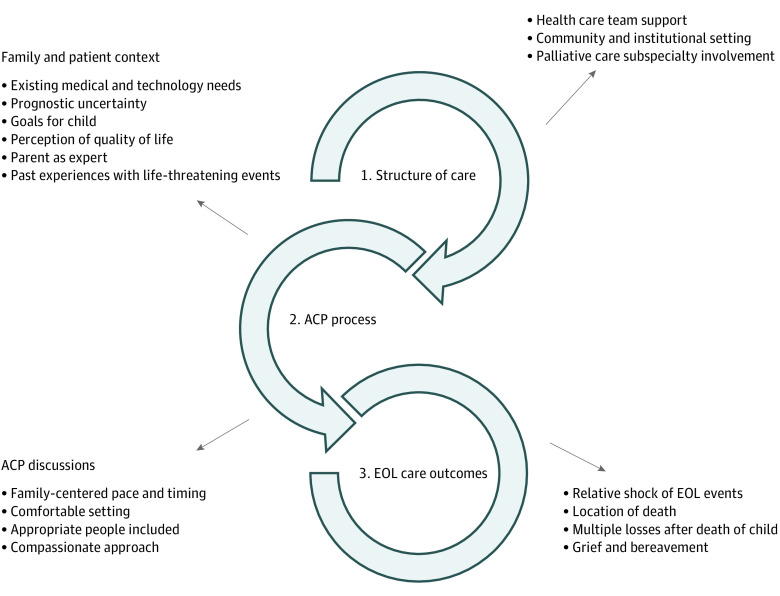
Advance Care Planning (ACP) Themes and Subthemes EOL indicates end of life.

**Table 2.** Example Quotations by Theme and Subtheme

Theme	Quotation (Participant identifier)
**Structure of care**	
Health care team support	“With someone who’s medically fragile, to have those members of the team that are going to be involved in the lifetime involved early, helps build the relationship, helps build the trust of the parents with the doctors, and that made all the difference in those last moments with [daughter].” (ACP040)
	“Through the Palliative Care team, I was able to have those hard conversations about what is it like to have [daughter]. I remember my first meeting when she was a year. We sat at this table with our doctor and stuff like that, and, [doctor] came in, and said, ‘Tell me about your daughter,’ and I said, ‘She’s got this wrong, she’s missing her eye. She has a this, and her lungs are bad, and she’s got scoliosis’. And he goes, ‘No, no, no. Tell me about [daughter]. Like what is she like?’ And I looked at my husband, and I just teared up and saw that I actually don’t know who she is. All I know is her illness.” (ACP040)
**ACP process**	
Family and patient context	
Existing medical and technology needs	“[Daughter] needed more and more equipment in the last year, cough-assist, a machine, and an oximeter, to watch her oxygen, so bringing those pieces of equipment to the house, for me, was natural.” (ACP040)
Prognostic uncertainty	“We never knew what was happening next. So we just, we kind of reacted instead of predicted.” (ACP008)
	“It was really hard to gauge where, whether [son] was going to give us 2 weeks or the extra year and a half that he did.” (ACP023)
Goals for child	“Our biggest goal for care, when he became sick, was for us to be able to do as much as of it as possible at home, to avoid long hospital stays.” (ACP024)
	“We had talked about taking [son] out to the aquarium … And it was like 1 of our small goals, because taking him out was very difficult. But when we were at [hospice], they arranged everything for us, we had a nurse come with us, taxi drive us, it was just, everything was well-planned and it was just, that gesture, that showed how thoughtful.” (ACP024)
Perception of quality of life	“Things got more challenging the last years, so we started having the [ACP] discussions within that team which was, really difficult. I initiated it again, when things just weren’t going as well and her quality of life was changing.” (ACP020)
	“You look at your child and yes there are tubes, and yes, they’re in a wheelchair or a special stroller, but what are their mannerisms? Are they smiling? Are they laughing? What’s the most quality they can have? Are they a happy child? We looked at her as a person. Don’t look at all the stuff you have to do to keep her alive; how is she doing?” (ACP028)
Parent as expert	“They have to listen to the parents, like I didn’t want her poked a million times. I knew where the best place was … I think things could have been done differently if they would have listened to me rather than just doing their job.” (ACP009)
	We spent a lot of time researching health fields that were involved with [daughter], like she had a lot of the different departments involved, and we were already really quite good with the maintenance of the G-J [gastro-jejunal] tube and the oxygen, we took care of all her meds and stuff as well.” (ACP039)
Past experiences with life-threatening events	“Well, a lot of times when we’re in those meetings, you’ve already suffered a traumatic experience in the hospital, if not 1 … 2, 3, or 4 [times].” (ACP028)
	“A lot of pneumonias, quite a few hospitalizations, and then, eventually, in the end, seizures became an issue, we were phoning 911 from the house and had to go a couple times, cause the rescue meds weren’t even doing anything.” (ACP041)
ACP discussions	
Family-centered pace and timing	“I wish we would have had it sooner and had maybe, smaller chunks of the conversation rather than just, we ended up having like 1 really big, intense conversation, and it was, it was a lot, and it felt like rushed.” (ACP009)
Comfortable setting	“I don’t think it was the right place at all. I think, and I can understand that sometimes it’s hard to get places, but it was in a small, tiny, exam room basically.… It was uncomfortable, with, you know, 2 adults, a stroller and a baby. And then, 3 other physicians, we had there. So, it was small and tight and cramped and it made the situation even more hard.” (ACP009)
	“The team was good at asking, hey do you want to book a room with this, or do you want to talk a little bit here, kind of thing.” (ACP040)
Appropriate people included	“Sometimes a different person coming in, you know, dealing with clients who don’t want to retell their story over and over again, so somebody could catch up in a way, so that consistency would, I mean, having a person who’s already been there, involved in the discussions was helpful for us.” (ACP023)
	“I was approached, I would say a good 4 times, to have discussions without my husband present. I was the one that took full reins on her care. But, for decisions that were made for her quality of life and her end-of-life care and all of that, I felt very uncomfortable that I was approached by myself. Some of the talks should not have happened without my husband present.” (ACP009)
Compassionate approach	“I think sometimes, it was, a lot of things were just said very matter-of-factly.… I understand physicians are dealing with this on an ongoing basis, daily, a lot. Some things were said, very much like, well, this is what, it was just, sometimes it needed a little more compassion and understanding for the fact that, our family is going through this and this is bigger than just, CPR.” (ACP009)
	“Everybody was very caring and very thorough in the way that they presented it to us. They were never annoyed, like, they were always happy to repeat if we didn’t understand something. It was a lot to take, as parents, and I feel that they really did take the time to walk us through it, and just to show some compassion, which was really nice.” (ACP024)
**End-of-Life care outcomes**	
Relative shock	“It came as, a shock, even though knowing that every day was a gift with [son], it came as a shock to me, because I didn’t realize how quickly things could happen.” (ACP041)
	“I said to him, do you think this is reversible, because she had been, up until that day, the moment that it happened, she had been doing really well.” (ACP020)
	“It was sudden in a sense that we weren’t expecting it; we were expecting to be leaving the PICU [pediatric intensive care unit] like any day, we were prepared to go [home]. She had seemed to be better, so, we weren’t prepared.” (ACP009)
Location	“I always thought that I want her to die at home because her family will be there and she’ll be more happy, and then, when it comes down to it, and you think about how that’s gonna affect everybody else, I just couldn’t do it and I’m so glad we did it where we were, cause all the symptoms she had, there’s no way I could have managed that at home on my own.” (ACP020)
	“As much as I pictured [daughter] dying in the hospital because of my medical mind… it worked at [hospice], and it was beautiful.” (ACP040)
Multiple losses	“[It’s] amazing the amount of people that were involved in her care. And now all those people are gone. And that’s super hard.” (ACP040)
	“Once your child dies, that team of doctors, that whole [hospital] was our home. It’s gone. All of a sudden, now, you’ve lost 2 families. And that’s, and then the third being your, the nurses that were in your home. It’s just empty. Everything’s gone. Your child’s gone. Your family, your, your medical family is gone.… And your community’s medical family is gone. And you’re alone. It’s like waking up, and it’s like everyone’s dead.” (ACP028)
Grief and bereavement	“The bereavement stuff is good, but I think there needs to be counseling. Mandatory counseling. That you can go to, so, it’s like a weaning off process, of weaning off the hospital. It’s giving you a piece of paper and telling you to find a counselor that is in the community who knows nothing about your 13-year experience? I still haven’t found anybody. I would much rather walk into [hospital] and talk with a counselor that’s gonna be there for end of life.” (ACP028)
	“I guess finding other families who have children with medical[ly] complex issues. How our experience is with [son], it’s hard to find someone to relate to.… In the previous circles that we have gone to, the grief programs, they’re grieving a child who died by suicide … or a child who got murdered. And then, we think how do I really relate my grief? So other medical[ly] complex, grief support, like having families that can relate.” (ACP023)

Abbreviation: ACP, advance care planning.

### Structure of Care

Parents described how the health care system and team structure during the child’s routine care and acute illnesses provided an important foundation on which ACP was situated (Table 2). Many stated that having trusted health care professionals who knew their child well was an important prerequisite for ACP. The presence of medical supports in their local community, such as physicians, home and school nursing, and the resources in their tertiary care hospital, including subspecialty teams and access to higher intensity care, were important factors that influenced ACP. For example, community and tertiary care resources influenced choices about location of care during times of illness or disease progression. Furthermore, many participants described the involvement of a subspecialty palliative care team as helpful for exploring goals of care.

### ACP Process

The ACP process was divided into 2 subthemes: (1) the family and patient context and (2) the ACP discussions. The family and patient context (Table 2) included an understanding of the child’s existing medical and technological needs, given that these often informed ACP decisions (eg, whether to add new technology, feasible locations of care in instances of worsening illness or for end-of-life care). Parents also described the degree of prognostic uncertainty, which was often high, as another aspect of their child’s unique situation that needed to be taken into account. Parents’ perceptions of their child’s quality of life and their specific goals for their children (both short-term and long-term) were key contributors to ACP. Examples included goals for being at home together as a family as much as possible or having typical family outings. Parents appreciated when their own expertise in their child’s care was acknowledged and valued, given that their knowledge was important for contributing to medical care decisions. Finally, CMC’s recurrent experiences with severe illness and life-threatening deteriorations were common and important influencers of ACP. Medical decisions regarding care escalation during an acute deterioration were influenced by the child’s past experiences with escalations in care under similar clinical circumstances. For example, parents spoke about prior experiences with escalations and what interventions were successful in stabilizing their child; this guided decisions about whether to embark on similar interventions in the future.

Parents’ reflections on their experiences with ACP discussions (Table 2) revealed that their preferences regarding pace and timing varied. Many felt that ACP discussions should occur early and continue regularly. Others expressed that they should be the ones to indicate readiness to engage in these conversations or felt that the conversations were too frequent. Having a comfortable setting, such as a quiet room with adequate seating, was important. Another theme was having the appropriate people present, including health care professionals who knew the patient and family well and key family caregiver(s) (eg, ensuring both parents were present). From the perspective of communication, the importance of health care professionals expressing compassion above all else was a common theme.

### End-of-Life Care Outcomes

Parents shared a number of reflections related to their experiences with end-of-life care (Table 2). None of the parents in our study had a child die at home. They felt more comfortable with their child dying in the hospital or hospice and reflected that ACP did help them consider different options for the location of end-of-life care. Parents noted that although their child experienced recurrent life-threatening events and they knew their child would likely have a shortened life span, the timing and circumstances around death often came as a shock. In addition to this, many parents described feeling not only the loss of their child but also the loss of the relationships they had built with individuals in their child’s large care team. Finally, several parents highlighted the need for improved grief and bereavement support following their child’s death, specifically in terms of having counselors and family support groups who understand the unique experience of caring for and losing a CMC.

## Discussion

This is a novel study of bereaved family caregivers’ experiences with ACP in that it focuses on CMC. The study adds to a growing body of literature in the area of pediatric ACP that no longer focuses only on typically developing adolescents who are capable of participating in their own ACP^14,15^ but also considers younger or noncommunicative children whose caregivers are faced with making decisions on their children’s behalf. In the case of CMC, caregivers often have to make those decisions in the context of a chronic disease course without a predictable trajectory. Perspectives from parents who have experienced the full life and death of their child are crucial for improving our understanding of optimal ACP for CMC.

The results of our study align with previous findings that a supportive relationship with health care professionals is key for effective ACP. A study by Mitchell et al^16^ that examined parental experiences with end-of-life decision-making for children in the intensive care setting also revealed the importance of having trusted health care professionals involved in ACP. In terms of the ACP process itself, the study by Mitchell et al^16^ also demonstrated the importance of parental knowledge as a key factor in ACP. Furthermore, demonstrating compassion in the discussion was identified in our and previous studies^17^ as being of utmost importance. As part of that compassionate approach, involving both parents and guardians in the discussion was preferred. In keeping with other centers,^18^ our study revealed that a high proportion of CMC whose families engaged in ACP had a pediatric palliative care team involvement.

Another theme that arose in our study was the optimal timing of ACP. The parent perspectives gathered in our study reflected that timing is important and that an earlier, step-wise conversation is appreciated by some, but there is no single approach that is preferred by all. In a similar study including health care professionals and parents of current CMC at our center,^9^ a flexible approach that was adaptable to parent needs was preferred. In another study of parental perceptions of ACP for children on long-term assisted ventilation,^19^ the issue of deciding when to start ACP discussions was challenging, and many parents preferred not to discuss advance directives during periods of stability. Others have found that parents prefer an individualized and gradual approach to ACP and a health care professional who has worked with the family consistently to facilitate those discussions.^5,7^ A dynamic and family-centered approach is likely needed, with the ability to initiate ACP outside of a time of crisis and then reflect with parents on the best way to discuss goals of care moving forward.

Several findings emerged from our study, including the themes related to the patient and family context, that help frame options for care moving forward. Parents consistently raised the child’s existing medical and technology supports as well as the degree of prognostic uncertainty as important factors of ACP. The family’s experiences with their child’s prior life-threatening events emerged repeatedly as a theme that played a key role in informing ACP. These aspects of the CMC patient and family experience may differ from other patients engaging in ACP, whose illness trajectory and end of life follow a more predictable trajectory. As such, they are important factors that clinicians should seek to understand when embarking on ACP with CMC.

Having lived through the entire disease trajectory and end of life of their child, family caregivers were able to reflect on the end-of-life care experience and how this was informed by ACP. Many parents described a feeling of relative shock when their child’s death actually happened, even though they had experienced multiple life-threatening events in the past. Despite knowing their child would likely die young and from similar causes, the final illness was often similar to clinical deteriorations in the past. We posit that there may be a degree of desensitization that occurs over time for parents who experience the up-and-down clinical course that is common for children with chronic medical fragility. Once they have faced a life-threatening situation several times, parents may adjust to their child surviving against the odds each time, resulting in a feeling of shock when the child ultimately does not survive.

When examining the bereavement experience, the feeling of experiencing multiple losses after the child’s death was shared strongly among participants. Parents described that this added sense of loss from the sudden absence of multiple health care professionals and community professionals who surrounded the child in their day-to-day care. Grief and bereavement support was desired not only to cope with the loss of the child but also to deal with this abrupt change and loss of relationships with the professionals who cared for their child and family.

### Limitations

This study has limitations. First, it took place at a single center. In the future, interviewing parents of CMC from other centers would be beneficial to gain broader perspectives. Although we reached thematic saturation, the available participant pool was small, given that many families’ contact information was not available. Although sampling began as purposive, all eligible families were ultimately contacted. Data on those we were unable to contact or who declined participation were not available. Our sampling only included the Complex Care and LTV clinics although there are some patients who meet criteria for CMC who were not treated in these clinics. It is possible that access to the multidisciplinary professionals in these clinics informed ACP. Interestingly, all CMC in our study experienced an in-hospital or hospice death, in contrast with the wider pediatric population in the center, in which a significant proportion of deaths occur at home. This in itself warrants further study. Additionally, we had participation almost exclusively from mothers, so a future study of fathers’ insights would be helpful.

## Conclusions

In this study, bereaved families’ experiences shed light on important aspects of ACP for CMC that warrant further study. CMC experience an unpredictable disease trajectory, and effective ACP discussions reflected this uncertainty. Parents appreciated a compassionate approach that involved trusted health care professionals who followed the pace and timing that were best for that particular family. Their experiences suggest that discussions should be informed by the child’s unique context and parental expertise regarding the child’s care. Understanding the common experiences with recurrent life-threatening events may be useful for providing anticipatory guidance. The added sense of loss that may be experienced by the family after the child’s death, when they no longer have the large circle of health care professionals providing support, is an important consideration for those providing grief support. The key concepts captured in this study can help inform future research and development of ACP processes for CMC.

## Notes

Supplement. **eTable. **Semistructured Interview Guide for Bereaved Family Caregivers

## References

[1] Himelstein BP, Hilden JM, Boldt AM, Weissman D Pediatric palliative care. N Engl J Med. 2004;350(17):1752-1762. doi:10.1056/NEJMra030334 15103002

[2] Judson L Global childhood chronic illness. Nurs Adm Q. 2004;28(1):60-66. doi:10.1097/00006216-200401000-00013 14986511

[3] Dewan T, Cohen E Children with medical complexity in Canada. Paediatr Child Health. 2013;18(10):518-522. doi:10.1093/pch/18.10.518 24497777 PMC3907346

[4] Cohen E, Kuo DZ, Agrawal R, Children with medical complexity: an emerging population for clinical and research initiatives. Pediatrics. 2011;127(3):529-538. doi:10.1542/peds.2010-0910 21339266 PMC3387912

[5] DeCourcey DD, Silverman M, Oladunjoye A, Wolfe J Advance care planning and parent-reported end-of-life outcomes in children, adolescents, and young adults with complex chronic conditions. Crit Care Med. 2019;47(1):101-108. doi:10.1097/CCM.0000000000003472 30303834

[6] Advance care planning for paediatric patients. Paediatr Child Health. 2008;13(9):791-805. doi:10.1093/pch/13.9.791 19436544 PMC2603159

[7] Lotz JD, Daxer M, Jox RJ, Borasio GD, Führer M “Hope for the best, prepare for the worst”: a qualitative interview study on parents’ needs and fears in pediatric advance care planning. Palliat Med. 2017;31(8):764-771. doi:10.1177/0269216316679913 27881828 PMC5557107

[8] Hammes BJ, Klevan J, Kempf M, Williams MS Pediatric advance care planning. J Palliat Med. 2005;8(4):766-773. doi:10.1089/jpm.2005.8.766 16128650

[9] Orkin J, Beaune L, Moore C, Toward an understanding of advance care planning in children with medical complexity. Pediatrics. 2020;145(3):e20192241. doi:10.1542/peds.2019-2241 32054820

[10] Beecham E, Oostendorp L, Crocker J, Keeping all options open: parents’ approaches to advance care planning. Health Expect. 2017;20(4):675-684. doi:10.1111/hex.12500 27670148 PMC5512998

[11] Wharton RH, Levine KR, Buka S, Emanuel L Advance care planning for children with special health care needs: a survey of parental attitudes. Pediatrics. 1996;97(5):682-687. 8628607

[12] Provincial Council of Maternal and Child Health CCKO standard operational definition CMC-MFTD. Published October 9, 2015. Accessed April 22, 2020. https://www.pcmch.on.ca/resource/ccko-operational-definition/pcmch-standard-operational-definition-cmc-mftd_ac-edits-v2-2/

[13] Cohen E, Berry JG, Camacho X, Anderson G, Wodchis W, Guttmann A Patterns and costs of health care use of children with medical complexity. Pediatrics. 2012;130(6):e1463-e1470. doi:10.1542/peds.2012-0175 23184117 PMC4528341

[14] Lyon ME, Jacobs S, Briggs L, Cheng YI, Wang J Family-centered advance care planning for teens with cancer. JAMA Pediatr. 2013;167(5):460-467. doi:10.1001/jamapediatrics.2013.943 23479062

[15] Dallas RH, Kimmel A, Wilkins ML, ; Adolescent Palliative Care Consortium. Acceptability of family-centered advanced care planning for adolescents with HIV. Pediatrics. 2016;138(6):e20161854. doi:10.1542/peds.2016-1854 27940700 PMC5127070

[16] Mitchell S, Spry JL, Hill E, Coad J, Dale J, Plunkett A Parental experiences of end of life care decision-making for children with life-limiting conditions in the paediatric intensive care unit: a qualitative interview study. BMJ Open. 2019;9(5):e028548. doi:10.1136/bmjopen-2018-028548 31072863 PMC6528052

[17] Zwakman M, Jabbarian LJ, van Delden J, Advance care planning: a systematic review about experiences of patients with a life-threatening or life-limiting illness. Palliat Med. 2018;32(8):1305-1321. doi:10.1177/0269216318784474 29956558 PMC6088519

[18] Harmoney K, Mobley EM, Gilbertson-White S, Brogden NK, Benson RJ Differences in advance care planning and circumstances of death for pediatric patients who do and do not receive palliative care consults: a single-center retrospective review of all pediatric deaths from 2012 to 2016. J Palliat Med. 2019;22(12):1506-1514. doi:10.1089/jpm.2019.0111 31233350 PMC6998041

[19] Edwards JD, Kun SS, Graham RJ, Keens TG End-of-life discussions and advance care planning for children on long-term assisted ventilation with life-limiting conditions. J Palliat Care. 2012;28(1):21-27. doi:10.1177/082585971202800104 22582468 PMC3682656

[20] Braun V, Clarke V Using thematic analysis in psychology. Qual Res Psychol. 2006;3(2):77-101. doi:10.1191/1478088706qp063oa

[21] Vaismoradi M, Turunen H, Bondas T Content analysis and thematic analysis: implications for conducting a qualitative descriptive study. Nurs Health Sci. 2013;15(3):398-405. doi:10.1111/nhs.12048 23480423

[22] Padgett DK Strategies for Rigor In: Qualitative Methods in Social Work Research. 2nd ed Sage Publications Inc; 2008:179-198.

[23] Tong A, Sainsbury P, Craig J Consolidated criteria for reporting qualitative research (COREQ): a 32-item checklist for interviews and focus groups. Int J Qual Health Care. 2007;19(6):349-357. doi:10.1093/intqhc/mzm042 17872937

[24] Donabedian A The quality of care—how can it be assessed? JAMA. 1988;260(12):1743-1748. doi:10.1001/jama.1988.03410120089033 3045356

[25] Sinuff T, Dodek P, You JJ, Improving end-of-life communication and decision making: the development of a conceptual framework and quality indicators. J Pain Symptom Manage. 2015;49(6):1070-1080. doi:10.1016/j.jpainsymman.2014.12.007 25623923

