# Supplementary material for: Assessment of Bereaved Caregiver Experiences of Advance Care Planning for Children With Medical Complexity
Source: JAMA Netw Open. 2020 Jul 28;3(7):e2010337. doi: 10.1001/jamanetworkopen.2020.10337 (PMC7388020; doi:10.1001/jamanetworkopen.2020.10337)
Supplement: Supplement. — eTable. Semistructured Interview Guide for Bereaved Family Caregivers [file jamanetwopen-3-e2010337-s001.pdf]

Supplementary Online Content

Lord S, Moore C, Beatty M, et al. Assessment of bereaved caregiver experiences of advance care planning for children with medical complexity. *JAMA Netw Open*. 2020;3(7):e2010337. doi:10.1001/jamanetworkopen.2020.10337

eTable. Semistructured Interview Guide for Bereaved Family Caregivers

This supplementary material has been provided by the authors to give readers additional information about their work.

© 2020 Lord S et al. *JAMA Network Open.*

eTable. Semistructured Interview Guide for Bereaved Family Caregivers

1. Perhaps you can start off by telling me about your child? Who was s/he as a person? And what were his/her medical challenges?

2. During your child's life, what were your hopes or goals for your child? *Prompt:* What was most important to you for your child (eg. Being at home or with family, going to school, developmental milestones, comfort)? Did these goals change over time? If so, what do you think caused them to change?

3. Can you tell me about any discussions about goals of care you had for your child? Prompt: These are discussions about how your goals for your child might have influenced health care decisions for him/her.

4. Goals of care discussions sometimes involve discussing the role of attempting life-prolonging interventions in someone with a serious illness (eg. CPR (compressing the chest, using equipment to breathe for a child, using medications to support blood pressure if a child's heart or breathing stops). Is this something that was ever discussed in the care of your child? *Prompt:* If so, how was it discussed? How could the team have improved the way this was brought up or discussed with you

5. Thinking back to any goals of care discussions you had, who was the primary health care provider that had these conversations with you? Where did they happen? *Prompt:* inpatient ward, outpatient clinic, ICU, other place? Was this the right place in your opinion? Was it with the right people?

1. How do you feel about the goals of care discussions you had for your child? *Prompt:* Do you think these started at the right time? Too early? Too late? Why do you feel this way? When do you think the "best" or "right" time is to have these discussions? What went well? What went poorly? How did these discussions make you feel? If they could be done in the best way possible, what would that look like?

7. What problems or challenges do you think existed around having goals of care discussions for your child? Any challenges on the part of your child's healthcare providers? What about within your family or yourself? Is there anything you wish could have been done differently?

8. What influenced you the most in your thinking about making major care decisions for your child? *Prompt*: Healthcare professionals? Family? Friends? Other? Do you have any examples?

9. Looking back, did your child's illness progress in a way you expected? *Prompts:* How did it differ from what you expected? Was there a time when you realized that the end of your child's life was coming soon, or was it very unexpected?

© 2020 Lord S et al. *JAMA Network Open.*

10. Can you tell me a bit about his/her end of life if you are comfortable? *Prompts:* Where was the location of your child's death? Were there options discussed for this location? Was this the place that was most right for your family? Which health care provider(s) had discussions about your child's end of life with you? Was talking about end of life something you had thought about before those discussions?

11. Did you feel that having goals of care discussions affected the end of life experience for your child and family? *Prompt*: location of end of life care, changes in medical interventions, opportunities for memory building/legacy creation. If so, was this in a positive way, a negative way, or both?

12. Are there things that were said or done during goals of care discussions that have left a lasting impression?

13. Are there any other things you would like to share about your wishes or expectations for these discussions? *Prompt:* Is there anything you would like to share about how the health care team could do a better job when discussing goals of care with patients and their families? How could we provide the best possible information and recommendations about planning for a child's future medical care?

14. Is there anything else you wish that care providers looking after children with complex medical needs could learn from your experience?

© 2020 Lord S et al. *JAMA Network Open.*
